# Decarboxylation of Pyruvate to Acetaldehyde for Ethanol Production by Hyperthermophiles

**DOI:** 10.3390/biom3030578

**Published:** 2013-08-21

**Authors:** Mohammad S. Eram, Kesen Ma

**Affiliations:** Department of Biology, University of Waterloo, 200 University Avenue West, Waterloo, Ontario N2L 3G1, Canada; E-Mail: smeram@uwaterloo.ca

**Keywords:** pyruvate decarboxylase, hyperthermophiles, alcohol fermentation, alcohol dehydrogenase, pyruvate ferredoxin oxidoreductase, pyruvate, acetaldehyde, ethanol

## Abstract

Pyruvate decarboxylase (PDC encoded by *pdc*) is a thiamine pyrophosphate (TPP)-containing enzyme responsible for the conversion of pyruvate to acetaldehyde in many mesophilic organisms. However, no *pdc*/PDC homolog has yet been found in fully sequenced genomes and proteomes of hyper/thermophiles. The only PDC activity reported in hyperthermophiles was a bifunctional, TPP- and CoA-dependent pyruvate ferredoxin oxidoreductase (POR)/PDC enzyme from the hyperthermophilic archaeon *Pyrococcus furiosus*. Another enzyme known to be involved in catalysis of acetaldehyde production from pyruvate is CoA-acetylating acetaldehyde dehydrogenase (AcDH encoded by *mhpF* and *adhE*). Pyruvate is oxidized into acetyl-CoA by either POR or pyruvate formate lyase (PFL), and AcDH catalyzes the reduction of acetyl-CoA to acetaldehyde in mesophilic organisms. AcDH is present in some mesophilic (such as clostridia) and thermophilic bacteria (e.g., *Geobacillus* and *Thermoanaerobacter*). However, no AcDH gene or protein homologs could be found in the released genomes and proteomes of hyperthermophiles. Moreover, no such activity was detectable from the cell-free extracts of different hyperthermophiles under different assay conditions. In conclusion, no commonly-known PDCs was found in hyperthermophiles. Instead of the commonly-known PDC, it appears that at least one multifunctional enzyme is responsible for catalyzing the non-oxidative decarboxylation of pyruvate to acetaldehyde in hyperthermophiles.

## 1. Introduction

Thermophilic microorganisms can be categorized into several groups: moderate thermophiles, or simply thermophiles, are those that grow optimally between 50–64 °C, extreme thermophiles are those with optimal growth temperatures between 65–79 °C. Finally, the organisms that can grow optimally above 80 °C are called hyperthermophiles. Hyperthermophiles can survive at room temperature for long periods of time, but cannot propagate at temperatures lower than 50 °C [1,2,3,4,5]. 

Hyperthermophilic microorganisms are widely studied for their remarkable scientific values and industrial potential. It is generally accepted that hyperthermophilic enzymes have very similar functions and catalytic mechanisms to their mesophilic ones. However, most of the hyperthermophilic enzymes characterized so far have optimum temperatures close to the host organism’s growth requirements; thus, due to their intrinsic properties, the enzymes are stable and active under conditions that are detrimental to their mesophilic counterparts. Interestingly, enzymes from such extremophiles usually show increased stability not to one, but to several environmental factors. There are a number of advantages for using the hyper/thermophilic enzymes (especially for industrial applications) over their mesophilic partners, including the reduced risk of contamination during industrial processes, the possibility of self-distillation of the products at high temperatures, decreased viscosity and increased solubility/bioavailability of both the enzyme and the substrate(s) leading to minimization of the diffusion limitations, and elimination of the costly transportation under cold temperature-controlled environment [6,7,8,9,10].

The aforementioned properties along with high demand from the biotech industries for the development of “tailor-made” bio-catalysts have created significant attention on the biochemistry and physiology of these organisms. Enzymes such as proteases, polymerases, hydrolases, isomerases, lipases, and oxidases are studied for their potential biotechnological exploitation, with the ultimate goal of using them or their products (mainly enzymatic) for biotechnological applications. 

It is also appealing to determine the molecular, biochemical, physiological, and evolutionary mechanisms that enable these organisms to adapt to such hostile environments. Furthermore, hyperthermophilic proteins serve as models to study enzyme evolution, structure-function relationships and catalytic mechanisms. The findings of these studies can benefit the design of highly stable and active enzymes to be used for many applications [6,11,12].

## 2. Microbial Production of Ethanol

Demand for biofuel as substitutes for oil-based fuels is increasing due to concerns related to national security, economic stability, environmental impacts, and global warming. The national research council of the United States has predicted that, by 2020, half of all organic chemicals and materials will be produced by bioconversion. Bio-ethanol can also be used as a precursor for many other commodity chemicals, such as acetaldehyde, acetic acid and their derivatives [13,14,15].

The most commonly used ethanologenic organisms being intensively studied or already in use for industrial-scale production are *Zymomonas* *mobilis*, *Saccharomyces* *cervisiae*, *Escherichia* *coli*, and *Klebsiella* *oxytoca*. Substantial attention and effort have been dedicated to redirecting the metabolic pathways of these and others towards higher yield of ethanol production, by means of metabolic engineering [16,17,18].

However, lack of suitable microorganisms that can efficiently convert the raw biomaterials to bio-ethanol has been one of the main obstacles to widespread use of bio-fuels. In addition to the ability to ferment a wide variety of sugars, some other features must be considered when choosing organisms for industrial-scale bio-ethanol production. These important features include but are not limited to the ability to have high ethanol yield, tolerance to fermentation products/by-products, simple growth requirements, and the ability to grow under conditions that prevent contaminating organisms from growing [14,16].

Production of bio-ethanol using thermophilic and hyperthermophilic organisms is the focus of many research groups. Extremophiles in general and hyperthermophiles in particular are outstanding organisms that produce highly stable enzymes due to their natural habitats, and many of them are able to tolerate changes in environment; making them good candidates for bio-ethanol production [19].

Several distinct advantages are associated with using thermophiles over mesophiles, including high temperatures and the mostly anaerobic nature of thermophilic organisms, which result in elimination of oxygenation and cooling of the fermenter. Another aspect is improved solubility of many reaction components at elevated temperatures [20]. In addition, the high temperature of the process leads to lowering the viscosity of reaction mixtures, causing improved production yields. Various thermophiles can ferment hexose and/or pentose sugars, as well as more complex substrates such as cellulose and xylan in some cases. Many of these organisms and their enzymes are relatively resistant to sudden pH or temperature changes and high concentrations of solvents [21,22,23]. High temperatures can result in lower gas solubility and significantly decrease the risk of process failure and product loss due to contamination that is the common problem in the yeast-based fermentation system. At the same time, high temperatures lower the cost of ethanol recovery due to the high volatility of ethanol at high fermentation temperatures [14,19,24,25]. However, there are some disadvantages associated with using hyperthermophiles, the most important one being their intrinsic low substrate tolerance and product/by-product inhibition [26,27]. Moreover, some of these organisms are mixed-fermenters that result in production of sometimes too many types of products during growth [14,16]. 

Application of metabolic engineering approaches has had a great impact on elimination of the problems associated with using thermophiles, and led to development of strains with bio-ethanol yields that are almost equal to those of the yeast-based systems. Members of the genus C*lostridium*, especially thermophilic members such as *Clostridium thermocellum*, have been studied intensively due to their competence in production substantial amounts of ethanol, butanol and hydrogen [24,28,29]. Members of the genus *Thermoanaerobacter*, including *T. ethanolicus*, *T.* *tengcongensis*, and *T. pentosaceus* are extremely thermophilic bacteria that are well studied for their high ethanol production potential especially from pentoses [30,31,32,33,34]. The genus *Geobacillus* has been studied widely for bio-ethanol production potential [19,29,35,36]. Production of ethanol, although at lower concentrations, has also been reported for the extremely thermophilic *Caldicellulosiruptor* species that includes* C. owensensis* [37], *C. kristjanssonii* [38], and *C. saccharolyticus* [39].

Compared to the thermophilic ethanol producers, very little is known about the ethanol production levels and pathways in the extremely thermophilic and hyperthermophilic microorganisms. It was shown that the peptide- and carbohydrate-fermenting hyperthermophilic archaeon *Pyrococcus furiosus* can produce H_2_, CO_2_, acetate, alanine, and small amounts of ethanol [40]. The strictly anaerobic archaeon *Thermococcus* sp. strain ES1 produced some ethanol and butanol when cultures were grown at low concentrations of elemental sulfur [41]. The production of ethanol as an end product of fermentation was also shown in the hyperthermophilic anaerobic archaeon *Thermococcus guaymasensis* [42] and more recently in the autotrophic hyperthermophile, *Thermococcus onnurineus* [43]. Within the bacterial hyperthermophiles, traces of ethanol have been reported in cultures of different Thermotogales including *T. hypogea* [44], *T. lettingae* [45], *T. neapolitana* [46], *Kosmotoga* *olearia* [47], and *Thermosipho affectus* [48].

## 3. Key Enzymes Involved in Ethanol Production

One of the key enzymes in both ethanol production pathways is alcohol dehydrogenase. Alcohol dehydrogenases are members of the oxidoreductase family and are present in all three domains of life [49,50]. They belong to the dehydrogenase/reductase superfamily of enzymes and catalyze the reversible inter-conversion of alcohols to corresponding aldehydes or ketons. ADHs can be classified based on their cofactor requirements: (I) the flavin adenine di-nucleotide (FAD)-dependent ADHs; (II) the pyrollo-quinoline quinone (PQQ), heme or cofactor F_420_ dependent ADHs; (III) NAD(P)-dependent ADHs [49,51]. Alternatively, they can be divided into three major groups based on their molecular size and metal contents: the first group is known as zinc-dependent long chain alcohol dehydrogenase; which have sizes of 300–900 amino acids, the second group is the short chain alcohol dehydrogenase: which contain no metal ions and have approximate lengths of 250 amino acids; and the third group is the long-chain iron dependent ADHs; with a length of 385–900 residues [49,50,51,52].

Many different ADHs have been characterized from various thermophilic and hyperthermophilic bacteria and archaea, with a majority of them being NAD(P)-dependent. Some of the more recently characterized hyper/thermophilic ADHs are those from *P.* *furiosus* [53,54], *Thermococcus hydrothermalis* [55], *Thermococcus kodakarensis* [56,57,58], *Thermococcus* *sibiricus* [59,60], *Thermococcus guaymasensis* [42], *Sulfolobus acidocaldarius* [61], *Thermococcus* strain ES1 [62], *Aeropyrum* *pernix* [63], *Thermotoga hypogea* [64], and *Pyrobaculum aerophilum* [65].

Although there is a relatively long list of ADHs isolated and characterized from thermophilic and hyperthermophilic archaea and bacteria, with the physiological roles of several proposed to be in the reduction of aldehydes to alcohols, other enzymes involved in the ethanol production pathways are not well characterized, especially the enzyme(s) that catalyze the production of acetaldehyde from pyruvate.

## 4. Pathways for the Production of Acetaldehyde from Pyruvate

**Figure 1:**
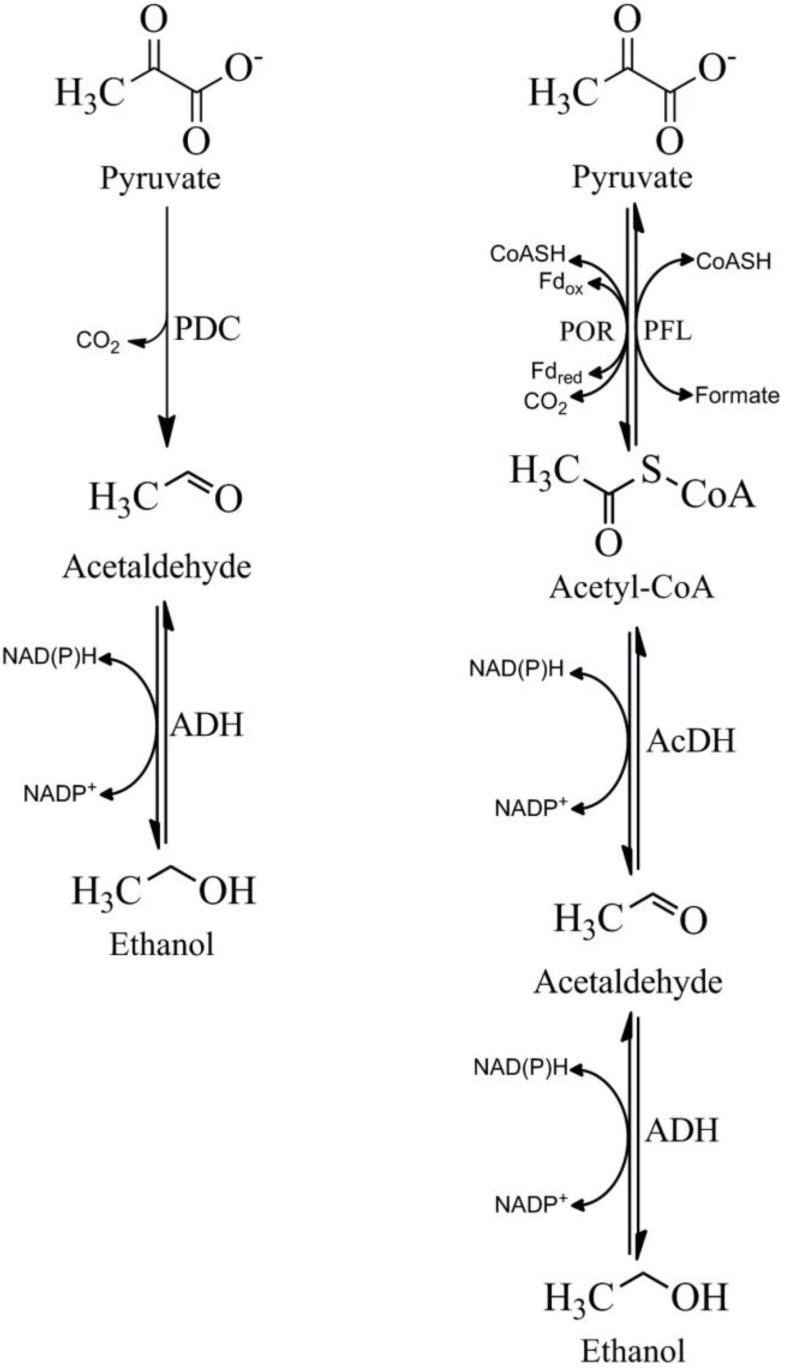
Two pathways of ethanol production from pyruvate. POR; Pyruvate ferredoxin oxidoreductase; PFL; Pyruvate formate lyase, AcDH; Acetaldehyde dehydrogenase, ADH; Alcohol dehydrogenase, PDC; pyruvate decarboxylase; CoASH; coenzyme A, Fd_ox_; oxidized ferredoxin, Fd_red_; reduced ferredoxin.

Pyruvate is an intermediate in the central metabolism of carbohydrates [66,67], and it can be converted to acetaldehyde that will eventually be reduced to ethanol using one of the following two pathways: 

(1) A two-step pathway that is used by yeast and a few bacteria like *Zymomonas mobilis* [68] and *Sarcina ventriculi* [69]. In this pathway pyruvate is non-oxidatively decarboxylated to acetaldehyde and carbon dioxide, which is catalyzed by pyruvate decarboxylase (PDC). Acetaldehyde is then converted to ethanol that is catalyzed by ADH (Figure 1);

(2) A three-step pathway that is more widespread in bacteria. Pyruvate is oxidatively decarboxylated to acetyl-coenzyme A (acetyl-CoA) by the metalloenzyme pyruvate ferredoxin oxidoreductase (POR) and/or pyruvate formate lyase (PFL). Acetyl-CoA is then converted to acetaldehyde by a CoA-dependent-acetylating acetaldehyde dehydrogenase (AcDH). Finally, acetaldehyde is reduced to ethanol by ADH. 

The key metabolite for the two known pathways is acetaldehyde. The thiamine pyrophosphate (TPP)-dependent enzyme pyruvate decarboxylase is the only enzyme proficient at direct conversion of pyruvate to acetaldehyde. Interestingly, a majority (but not all) of the enzymes which are involved in the acetaldehyde production pathways are members of the superfamily of TPP-dependent enzymes, which includes PDC, POR, and PFL [70,71].

TPP, also known as thiamine diphosphate (ThDP), is composed of an aromatic methylaminopyrimidine ring, linked to a methyl thiazolium ring via. a methylene group with a pyrophosphate group attached to a hydroxylethyl side chain. TPP is derived from the water-soluble vitamin B1 and is the most common cofactor for enzymes that catalyze the cleavage and formation of carbon-carbon bonds next to a carbonyl group; hence TPP-dependent enzymes are involved in a wide range of metabolic pathways. Unlike many other cofactors (e.g., nicotinamide adenine dinucleotide, NADH) which are basically co-reactants, TPP remains at the enzymes’ catalytic center and is directly involved in the catalysis of the reaction [72]. The reactions catalyzed by TPP-dependent enzymes can be divided into at least three groups: the oxidative reactions, non-oxidative reactions, and carboligation reactions [73].

## 5. Pyruvate Decarboxylase (PDC)

In 1911, Neuberg and Karczag for the first time described the decarboxylation of pyruvate to acetaldehyde in *S.* *cerevisiae*. In 1922, the same research group detected the potential of yeast in the formation of C-C bonds. Neuberg named the new enzyme “carboligase”, and assumed it to exist apart from “α-carboxylase” (PDC) in yeast [74]. However, the preliminary characterization of the enzymes’ cofactor was delayed until 1937, when Lohmann and Schuster analyzed structure of the enzymes’ cofactor to be “cocarboxylase” or “aneurinpyrophosphate” or thiamin diphosphate [73].

The enzyme catalyzes non-oxidative decarboxylation of α-keto acids to produce a corresponding aldehyde and carbon dioxide. The most extensively examined enzymes of this group are the ones from *Saccharomyces cerevisiae* and its bacterial counterpart *Z. mobilis*. In addition to decarboxylation of pyruvate, PDC also catalyzes the enantio-selective formation of 2-hydroxy ketons via. carboligase side reactions. 

PDC, or its gene (*pdc*), is found to be widely distributed in fungi and higher plants but it is relatively rare in prokaryotes and unknown in animals. In fungi, PDC is found in *Saccharomyces* *cerevisiae*, *Saccharomyces* *carlsbergensis* (also known as *S. pastorianus*) and *Saccharomyces* *uvarum*, *Neurospora* *crassa*, members of the *Kluyveromyces* species, members of the *Aspergillus* species, *Hanseniaspora* *uvarum*, *Schizosaccharomyces* *pombe*, and in *Candida* (*Torulopsis*) *glabrata*. PDC is present in a variety of plants, including maize (*Zea* *maize*), parsnip, orange, pea (*Pisum* *sativum*), jack bean, sweet potato, wheat, cotton wood, soybean and rice (*Oryza* *sativa*). In prokaryotes, PDC is found and studied in *Z. mobilis*, *Sarcina ventriculi*, *Clostridium* *botulinum*, *Acetobacter* species, *Zymobacter* *palmae*, and in *Erwinia* *amylovora* [75,76,77,78,79]. So far there has been no report on finding PDC/*pdc* homolog in thermophilic or hyperthermophilic bacteria or in any of the members of the third major evolutionary lineage of life, archaea as a whole [76,77,78,80].

PDCs from different organisms show at least a 30% identity at the amino acid level and most of them are composed of subunits of 562–610 amino acid residues. The holoenzyme is usually composed of four identical or non-identical subunits of approximately 60 kDa (ensuing in a total mass of about 240 kDa) in which every two subunits binds tightly (but not covalently) to a set of cofactors including TPP and Mg^2+^ ion. PDCs with four subunits are often arranged as a dimer of dimers, with multiple close contacts within the dimers and several contacts between the dimers. The contact area between two related dimers forms the “V” conformation that is a common property of all TPP-dependent enzymes studied so far, and it also has an essential role in cofactor binding for this group of enzymes [81,82].

The catalytic mechanism of PDC for the most part follows the principles of catalytic mechanisms of other TPP-dependent enzymes: in brief, carbonyl addition of pyruvate to the reactive C2 atom of the cofactor thiazolium ring [73] yields the intermediate 2-(2-lactyl)-TDP (LTDP). The subsequent release of carbon dioxide produces resonating carbanion/enamine forms of 2-(1-hydroxyethyl)-TDP (HETDP, also known as hydroxyethylidene-TPP). The resonating form is considered to be a central and highly reactive intermediate state in TPP-dependent enzymes acting on pyruvate. However, unlike most other TPP-dependent enzymes in which the intermediate is oxidized, the carbanion/enamine in PDC is protonated at the C2α position, yielding C2α-hydroxylethylthiamine diphosphate (HETDP) before the final release of acetaldehyde completes the reaction [72,83,84].

Crystal structures of several pyruvate decarboxylases are solved particularly from yeasts and *Z. mobilis* [81,85,86,87]. The active sites of these enzymes are also studied comprehensively using site-directed mutagenesis [88,89,90].

## 6. Pyruvate Ferredoxin Oxidoreductase (POR)

The enzyme pyruvate ferredoxin oxidoreductase (also known as pyruvate synthase as the reaction is reversible) is one of the best studied members of the 2-oxoacid oxidoreductase family [91,92,93]. The enzyme catalyzes coenzyme A and TPP-dependent oxidative decarboxylation of pyruvate to acetyl-CoA, releasing a molecule of CO_2_ and transferring the reducing equivalents to the electron acceptor ferredoxin or flavodoxin. Alternatively, in other pyruvate oxidizing enzymes, the reducing equivalents are transferred to NAD^+^ (in the case of pyruvate dehydrogenase using lipoate as oxidizing agent for the production of acetyl-CoA), to molecular-oxygen-producing hydrogen peroxide (in the case of pyruvate oxidase), or to the carbonyl groups producing formate (in case of pyruvate formate lyase) [94,95,96]. In acetaldehyde- and ethanol-producing organisms, acetyl-CoA is usually converted to acetaldehyde via. the CoA-dependent (acetylating) acetaldehyde dehydrogenase. 

POR uses iron-sulfur cluster chemistry to catalyze the pyruvate decarboxylation and release of acetyl-CoA. POR is an ancient molecule, and it seems to have existed even before the divergence of the domains of the bacteria and archaea [97]. The enzyme is present in all three domains of life. All archaea catalyze the conversion of pyruvate to acetyl-CoA using POR, and all of the archaeal genomes sequenced so far contain hetero-tetrameric PORs, which have been proposed to be the closest to the POR common ancestor [97,98]. 

POR is prevalent mainly in anaerobic bacteria and infrequently found in anaerobic protozoa, for example, in *Giardia duodenalis* [99] and *Enthamoeba* *histolytica* [100,101]. The enzyme has been isolated and studied from many different anaerobic or microaerophilic microorganisms including anaerobic bacteria like the genera *Clostridium* [102], *Moorella thermoacetica* [103] and anaerobic sulphate-reducing bacteria *Desulfovibrio africanus* [104,105,106,107]. In hyperthermophiles, PORs are characterized from the hyperthermophilic bacterium *Thermotoga maritima* [108] and hyperthermophilic archaea *Pyrococcus furiosus* [109] and *Archaeoglobus fulgidus* [110], as well as the methanogenic archaea *Methanosarcina barkeri* [111,112] and *Methanobacterium thermoautotrophicum* [113].

The quaternary oligomeric structure of the POR is variable depending on the source microorganism and can be homo-dimeric (e.g., most bacterial PORs), hetero-dimeric (e.g., POR of *Halobacterium salinarium*), hetero-tetrameric (archaeal PORs), and heteropentameric (anabolic PORs), although all of the PORs studied so far, regardless of their source and structure, seem to be phylogenetically related and derived from a common archaeal-type heterotetrameric ancestor [97,98].

The crystal structures of several POR have been determined. PORs from *Desulfovibrio africanus* (with and without bound substrate) and *Desulfovibrio vulgaris* [114,115,116] are among the most extensively studied PORs. POR is a metalloenzyme and all PORs studied so far contained between one and three [4Fe-4S] clusters arranged in a spatial order from the TPP located at the active center of the enzyme toward its surface, suggesting that they are part of an electron transfer pathway [117,118].

POR can also catalyze the reaction to form pyruvate from acetyl-CoA and carbon dioxide, which is the basis of the carbon dioxide fixation in many autotrophic microorganisms [119]. This type represents the so-called “anabolic” PORs that are studied from the thermophilic facultative aerobic bacterium *Hydrogenobacter* *thermophilus* [120,121,122], as well as the hydrogenotrophic methanoarchaeon *Methanococcus maripaludis* [123,124]. Interestingly, in the case of the heteropentameric POR of *M. maripaludis*, four subunits are very closely related to the archaeal heterotetrameric (ancestral) PORs, the fifth subunit has no known homologue within PORs. 

The general steps of the POR catalytic reactions follow the same principles as those of other TPP-dependent enzymes. However, the enzyme is unique in one aspect: unlike most other TPP-dependent enzymes, POR takes advantage of free radical chemistry to catalyze the decarboxylation reaction [94,125].

Pyruvate dehydrogenase complexes (PDH), which also catalyze the oxidative decarboxylation of pyruvate to acetyl-CoA using NAD^+^ as an electron acceptor, are normally present in aerobic organisms [126,127], which have been found in some thermopiles [128,129,130]. But no PDH has been identified in hyperthermophiles [131]. Since PDH does not play any significant role in the production of acetaldehyde and ethanol, no further description of this enzyme complex will be given in this review.

## 7. POR/PDC Bi-Functional Enzyme

In 1997, it was reported that the POR was also capable of converting pyruvate to acetaldehyde in the hyperthermophilic anaerobic archaeon *Pyrococcus furiosus* [80]. Unlike the commonly-known PDCs, which employ chemical rather than radical intermediates and therefore are oxygen insensitive, the reported PDC activity was highly oxygen sensitive. Both the POR and PDC activities of the hyperthermophilic enzyme were TPP- and coenzyme A-dependent. By using the coenzyme A analogue (desulfocoenzyme A), it was shown that coenzyme A has only a structural, and not a catalytic role in the catalyzed PDC reaction. Consequently, a “switch” mechanism was proposed for the enzyme’s bi-functionality, suggesting the conversion of active aldehyde to either acetyl-CoA or acetaldehyde, depending on the binding of CoA. According to the proposed model, binding of coenzyme A causes conformational changes in the intermediate structure, causing its protonation and generation of hydroxyethyl-TPP (HETDP). This reaction leads to release of acetaldehyde, allowing for the regeneration of TPP and possible release of CoA [80]. Ferredoxin is not required for its full PDC activity nor has any inhibitory effect when tested under *in vitro* conditions. However, it is likely that the *in vivo* PDC activity might be dependent on the availability of oxidized ferredoxin, which means that a lower ratio of oxidized to reduced ferredoxin may favor the PDC activity and *vice versa*. Therefore, it can be predicted that more acetaldehyde would be produced for being reduced to ethanol if the ratio of the oxidized to reduced ferredoxin would be kept very low under the anaerobic growth condition. Such a low ratio may also require a relatively low activity of the ferredoxin-oxidizing hydrogenases present in *P. furiosus* [132,133].

To date there has been no further study on the bi-functionality of the POR enzyme or the physiological relevance of such bifunctionality in any other organisms. It is not clear whether this bi-functionality is only a trait of *Pyrococcales*’ POR or a common property of all hyper/thermophilic PORs.

## 8. Acetaldehyde Dehydrogenase (CoA-Acetylating)

Acetaldehyde dehydrogenase (CoA-acetylating, EC 1.2.1.10) is a member of a very divergent superfamily of enzymes known as the “aldehyde dehydrogenases”. The prototype enzyme (*adhE*) was first discovered in *Escherichia coli* and is required for its anaerobic growth [134]. It was then discovered in the strictly anaerobic bacterium *Clostridium* *kluyveri* [135]. The enzyme is responsible for the conversion of acetyl-coenzyme A (acetyl-CoA) to acetaldehyde that is eventually converted to ethanol. Two forms of the enzyme are available: one is the monofunctional enzyme with only AcDH activity (*mhpF*) and the other is the bifunctional enzyme with both AcDH and ADH activities (*adhE*). The latter group is composed of an ADH active C-terminal and an AcDH active N-terminal, a structure believed to be the result of gene fusion between the genes encoding for each single enzyme [136,137].

Reports are available on isolation and characterization of the bifunctional NADP-dependent alcohol/acetaldehyde dehydrogenase (CoA-acetylating) from mesophilic microorganisms including *Giardia lamblia* [138] and *Enthamoeba* *histolytica* [101,139]. They are also present in some thermophiles, including *T. ethanolicus* [140,141], *T. mathranii* [32] and members of the genus *Geobacillus* [19]. However, no mono- or bi-functional AcDH activity was characterized from hyperthermophiles. Survey of the fully sequenced genomes of hyperthermophilic archaea and bacteria has shown no* adhE* or *mhpF* homologue either (Eram and Ma, unpublished data).

## 9. Conclusions

Many hyperthermophilic microorganisms produce ethanol as an end metabolic product. Although alcohol dehydrogenase and pyruvate ferredoxin oxidoreductase are found to be present, enzymes catalyzing the production of acetaldehyde from pyruvate are not well characterized. The commonly-known pyruvate decarboxylase and coenzyme A-dependent aldehyde dehydrogenase have not been identified. The only report of a bi-functional pyruvate decarboxylase is the POR/PDC from *P. furiosus*, which is thermostable but oxygen-sensitive. Therefore, it is likely that they use a two-step pathway to convert pyruvate to ethanol. The regulation of each of the POR/PDC activities is not clear but it may be related to the redox states inside the cells. To date, it appears that at least one multifunctional enzyme is responsible for catalyzing the non-oxidative decarboxylation of pyruvate to acetaldehyde in hyperthermophiles, and further study is needed to understand the catalysis of acetaldehyde production from pyruvate at high temperatures.

## References

[1] Wiegel J. (1990). Temperature spans for growth: Hypothesis and discussion. FEMS Microbiol. Lett..

[2] Charlier D., Droogmans L. (2005). Microbial Life at high temperature, the challenges, the strategies. Cell. Mol. Life Sci..

[3] Stetter K. (2006). History of discovery of the first hyperthermophiles. Extremophiles.

[4] Lebedinsky A., Chernyh N., Bonch-Osmolovskaya E. (2007). Phylogenetic systematics of microorganisms inhabiting thermal environments. Biochemistry (Mosc.).

[5] Wagner I.D., Wiegel J. (2008). Diversity of Thermophilic Anaerobes. Ann. NY Acad. Sci..

[6] Morozkina E., Slutskaya E., Fedorova T., Tugay T., Golubeva L., Koroleva O. (2010). Extremophilic microorganisms: Biochemical adaptation and biotechnological application. Appl. Biochem. Microbiol..

[7] Sommer P., Georgieva T., Ahring B.K. (2004). Potential for using thermophilic anaerobic bacteria for bioethanol production from hemicellulose. Biochem. Soc. Trans..

[8] Vieille C., Zeikus G.J. (2001). Hyperthermophilic enzymes: Sources, uses, and molecular mechanisms for thermostability. Microbiol. Mol. Biol. Rev..

[9] Haki G.D., Rakshit S.K. (2003). Developments in industrially important thermostable enzymes: A review. Bioresour. Technol..

[10] Egorova K., Antranikian G. (2005). Industrial relevance of thermophilic Archaea. Curr. Opin. Microbiol..

[11] Van den Burg B. (2003). Extremophiles as a source for novel enzymes. Curr. Opin. Microbiol..

[12] Atomi H., Sato T., Kanai T. (2011). Application of hyperthermophiles and their enzymes. Curr. Opin. Biotechnol..

[13] Lynd L.R., Wyman C.E., Gerngross T.U. (1999). Biocommodity engineering. Biotechnol. Prog..

[14] Zaldivar J., Nielsen J., Olsson L. (2001). Fuel ethanol production from lignocellulose: A challenge for metabolic engineering and process integration. Appl. Microbiol. Biotechnol..

[15] Mabee W.E., Saddler J.N. (2010). Bioethanol from lignocellulosics: Status and perspectives in Canada. Bioresour. Technol..

[16] Dien B.S., Cotta M.A., Jeffries T.W. (2003). Bacteria engineered for fuel ethanol production: Current status. Appl. Microbiol. Biotechnol..

[17] Buschke N., Schäfer R., Becker J., Wittmann C. (2012). Metabolic engineering of industrial platform microorganisms for biorefinery applications-optimization of substrate spectrum and process robustness by rational and evolutive strategies. Bioresour. Technol..

[18] Jang Y.-S., Park J.M., Choi S., Choi Y.J., Seung D.Y., Cho J.H., Lee S.Y. (2012). Engineering of microorganisms for the production of biofuels and perspectives based on systems metabolic engineering approaches. Biotechnol. Adv..

[19] Taylor M.P., Eley K.L., Martin S., Tuffin M.I., Burton S.G., Cowan D.A. (2009). Thermophilic ethanologenesis: Future prospects for second-generation bioethanol production. Trends Biotechnol..

[20] Bustard M.T., Burgess J.G., Meeyoo V., Wright P.C. (2000). Novel opportunities for marine hyperthermophiles in emerging biotechnology and engineering industries. J. Chem. Technol. Biotechnol..

[21] Huber H., Stetter K.O. (1998). Hyperthermophiles and their possible potential in biotechnology. J. Biotechnol..

[22] Schiraldi C., de Rosa M. (2002). The production of biocatalysts and biomolecules from extremophiles. Trends Biotechnol..

[23] Hough D.W., Danson M.J. (1999). Extremozymes. Curr. Opin. Chem. Biol..

[24] Lamed R., Zeikus J.G. (1980). Ethanol production by thermophilic bacteria: Relationship between fermentation product yields of and catabolic enzyme activities in *Clostridium thermocellum* and *Thermoanaerobium brockii*. J. Bacteriol..

[25] Klapatch T.R., Hogsett D.A.L., Baskaran S., Pal S., Lynd L.R. (1994). Organism development and characterization for ethanol production using thermophilic bacteria. Appl. Biochem. Biotechnol..

[26] Chang T., Yao S. (2011). Thermophilic, lignocellulolytic bacteria for ethanol production: Current state and perspectives. Appl. Microbiol. Biotechnol..

[27] Zeikus J.G., Ben-Bassat A., Ng T.K., Lamed R.J., Hollaender A., Rabson R., Rogers P., Pietro A.S., Valentine R., Wolfe R. (1981). Thermophilic ethanol fermentations. Trends in the Biology of Fermentations for Fuels and Chemicals.

[28] Demain A.L., Newcomb M., Wu J.H.D. (2005). Cellulase, clostridia, and ethanol. Microbiol. Mol. Biol. Rev..

[29] Barnard D., Casanueva A., Tuffin M., Cowan D. (2010). Extremophiles in biofuel synthesis. Environ. Technol..

[30] Tomás A.F., Karagöz P., Karakashev D., Angelidaki I. (2013). Extreme thermophilic ethanol production from rapeseed straw: Using the newly isolated *Thermoanaerobacter pentosaceus* and combining it with *Saccharomyces cerevisiae* in a two-step process. Biotechnol. Bioeng..

[31] Shaw A.J., Podkaminer K.K., Desai S.G., Bardsley J.S., Rogers S.R., Thorne P.G., Hogsett D.A., Lynd L.R. (2008). Metabolic engineering of a thermophilic bacterium to produce ethanol at high yield. Proc. Natl. Acad. Sci. USA.

[32] Yao S., Mikkelsen M.J. (2010). Identification and overexpression of a bifunctional aldehyde/alcohol dehydrogenase responsible for ethanol production in *Thermoanaerobacter mathranii*. J. Mol. Microbiol. Biotechnol..

[33] Svetlitchnyi V., Kensch O., Falkenhan D., Korseska S., Lippert N., Prinz M., Sassi J., Schickor A., Curvers S. (2013). Single-step ethanol production from lignocellulose using novel extremely thermophilic bacteria. Biotechnol. Biofuels.

[34] Yao S., Mikkelsen M. (2010). Metabolic engineering to improve ethanol production in *Thermoanaerobacter mathranii*. Appl. Microbiol. Biotechnol..

[35] Thompson A., Studholme D., Green E., Leak D. (2008). Heterologous expression of pyruvate decarboxylase in *Geobacillus thermoglucosidasius*. Biotechnol. Lett..

[36] Cripps R.E., Eley K., Leak D.J., Rudd B., Taylor M., Todd M., Boakes S., Martin S., Atkinson T. (2009). Metabolic engineering of *Geobacillus thermoglucosidasius* for high yield ethanol production. Metab. Eng..

[37] Huang C.-Y., Patel B.K., Mah R.A., Baresi L. (1998). *Caldicellulosiruptor owensensis* sp. nov., an anaerobic, extremely thermophilic, xylanolytic bacterium. Int. J. Syst. Bacteriol..

[38] Bredholt S., Sonne-Hansen J., Nielsen P., Mathrani I.M., Ahring B.K. (1999). *Caldicellulosiruptor kristjanssonii* sp. nov., a cellulolytic, extremely thermophilic, anaerobic bacterium. Int. J. Syst. Bacteriol..

[39] Van Niel E.W.J., Claassen P.A.M., Stams A.J.M. (2003). Substrate and product inhibition of hydrogen production by the extreme thermophile, *Caldicellulosiruptor saccharolyticus*. Biotechnol. Bioeng..

[40] Kengen S., de Bok F., van Loo N., Dijkema C., Stams A., de Vos W. (1994). Evidence for the operation of a novel Embden-Meyerhof pathway that involves ADP-dependent kinases during sugar fermentation by *Pyrococcus furiosus*. J. Biol. Chem..

[41] Ma K., Loessner H., Heider J., Johnson M., Adams M. (1995). Effects of elemental sulfur on the metabolism of the deep-sea hyperthermophilic archaeon *Thermococcus* strain ES-1: Characterization of a sulfur-regulated, non-heme iron alcohol dehydrogenase. J. Bacteriol..

[42] Ying X., Ma K. (2011). Characterization of a zinc-containing alcohol dehydrogenase with stereoselectivity from the hyperthermophilic archaeon *Thermococcus guaymasensis*. J. Bacteriol..

[43] Moon Y.-J., Kwon J., Yun S.-H., Lim H.L., Kim M.-S., Kang S.G., Lee J.-H., Choi J.-S., Kim S.L., Chung Y.-H. (2012). Proteome analyses of hydrogen-producing hyperthermophilic archaeon *Thermococcus onnurineus* NA1 in different one-carbon substrate culture conditions. Mol. Cell. Proteomics.

[44] Fardeau M.L., Ollivier B., Patel B.K.C., Magot M., Thomas P., Rimbault A., Rocchiccioli F., Garcia J.L. (1997). *Thermotoga hypogea* sp. nov., a xylanolytic, thermophilic bacterium from an oil-producing well. Int. J. Syst. Bacteriol..

[45] Balk M., Weijma J., Stams A.J.M. (2002). *Thermotoga lettingae* sp. nov., a novel thermophilic, methanol-degrading bacterium isolated from a thermophilic anaerobic reactor. Int. J. Syst. Evol. Microbiol..

[46] De Vrije T., Bakker R., Budde M., Lai M., Mars A., Claassen P. (2009). Efficient hydrogen production from the lignocellulosic energy crop *Miscanthus* by the extreme thermophilic bacteria *Caldicellulosiruptor saccharolyticus* and *Thermotoga neapolitana*. Biotechnol. Biofuels.

[47] DiPippo J.L., Nesbo C.L., Dahle H., Doolittle W.F., Birkland N.-K., Noll K.M. (2009). *Kosmotoga olearia* gen. nov., sp. nov., a thermophilic, anaerobic heterotroph isolated from an oil production fluid. Int. J. Syst. Evol. Microbiol..

[48] Podosokorskaya O.A., Kublanov I.V., Reysenbach A.L., Kolganova T.V., Bonch-Osmolovskaya E.A. (2011). *Thermosipho affectus* sp. nov., a thermophilic, anaerobic, cellulolytic bacterium isolated from a Mid-Atlantic Ridge hydrothermal vent. Int. J. Syst. Evol. Microbiol..

[49] Reid M.F., Fewson C.A. (1994). Molecular characterization of microbial alcohol dehydrogenases. Crit. Rev. Microbiol..

[50] Littlechild J.A., Guy J.E., Isupov M.N. (2004). Hyperthermophilic dehydrogenase enzymes. Biochem. Soc. Trans..

[51] Radianingtyas H., Wright P.C. (2003). Alcohol dehydrogenases from thermophilic and hyperthermophilic archaea and bacteria. FEMS Microbiol. Rev..

[52] Korkhin Y., Kalb A.J., Peretz M., Bogin O., Burstein Y., Frolow F. (1998). NADP-dependent bacterial alcohol dehydrogenases: Crystal structure, cofactor-binding and cofactor specificity of the ADHs of *Clostridium beijerinckii* and *Thermoanaerobacter brockii*. J. Mol. Biol..

[53] Machielsen R., Uria A.R., Kengen S.W.M., van der Oost J. (2006). Production and characterization of a thermostable alcohol dehydrogenase that belongs to the aldo-keto reductase superfamily. Appl. Environ. Microbiol..

[54] Van der Oost J., Voorhorst W.G.B., Kengen S.W.M., Geerling A.C.M., Wittenhorst V., Gueguen Y., de Vos W.M. (2001). Genetic and biochemical characterization of a short-chain alcohol dehydrogenase from the hyperthermophilic archaeon *Pyrococcus furiosus*. Eur. J. Biochem..

[55] Antoine E., Rolland J.-L., Raffin J.-P., Dietrich J. (1999). Cloning and over-expression in *Escherichia coli* of the gene encoding NADPH group III alcohol dehydrogenase from *Thermococcus hydrothermalis*. Eur. J. Biochem..

[56] Bashir Q., Rashid N., Jamil F., Imanaka T., Akhtar M. (2009). Highly thermostable l-threonine dehydrogenase from the hyperthermophilic archaeon *Thermococcus kodakaraensis*. J. Biochem. (Tokyo).

[57] Bowyer A., Mikolajek H., Stuart J.W., Wood S.P., Jamil F., Rashid N., Akhtar M., Cooper J.B. (2009). Structure and function of the l-threonine dehydrogenase (TkTDH) from the hyperthermophilic archaeon *Thermococcus kodakaraensis*. J. Struct. Biol..

[58] Wu X., Zhang C., Orita I., Imanaka T., Fukui T., Xing X.-H. (2013). Thermostable alcohol dehydrogenase from *Thermococcus kodakarensis* KOD1 for enantioselective bioconversion of aromatic secondary alcohols. Appl. Environ. Microbiol..

[59] Stekhanova T.N., Mardanov A.V., Bezsudnova E.Y., Gumerov V.M., Ravin N.V., Skryabin K.G., Popov V.O. (2010). Characterization of a thermostable short-chain alcohol dehydrogenase from the hyperthermophilic archaeon *Thermococcus sibiricus*. Appl. Environ. Microbiol..

[60] Lyashenko A.V., Bezsudnova E.Y., Gumerov V.M., Lashkov A.A., Mardanov A.V., Mikhailov A.M., Polyakov K.M., Popov V.O., Ravin N.V., Skryabin K.G. (2010). Expression, purification and crystallization of a thermostable short-chain alcohol dehydrogenase from the archaeon *Thermococcus sibiricus*. Acta Crystallogr. F Struct. Biol. Cryst. Commun..

[61] Pennacchio A., Giordano A., Pucci B., Rossi M., Raia C. (2010). Biochemical characterization of a recombinant short-chain NAD(H)-dependent dehydrogenase/reductase from *Sulfolobus acidocaldarius*. Extremophiles.

[62] Ying X., Grunden A., Nie L., Adams M., Ma K. (2009). Molecular characterization of the recombinant iron-containing alcohol dehydrogenase from the hyperthermophilic Archaeon, *Thermococcus* strain ES1. Extremophiles.

[63] Guy J.E., Isupov M.N., Littlechild J.A. (2003). The structure of an alcohol dehydrogenase from the hyperthermophilic archaeon *Aeropyrum pernix*. J. Mol. Biol..

[64] Ying X., Wang Y., Badiei H., Karanassios V., Ma K. (2007). Purification and characterization of an iron-containing alcohol dehydrogenase in extremely thermophilic bacterium *Thermotoga hypogea*. Arch. Microbiol..

[65] Vitale A., Thorne N., Lovell S., Battaile K.P., Hu X., Shen M., D’Auria S., Auld D.S. (2013). Physicochemical characterization of a thermostable alcohol dehydrogenase from *Pyrobaculum aerophilum*. PLoS One.

[66] Verhees C.H., Kengen S.W.M., Tuininga J.E., Schut G.J., Adams M.W.W., de Vos W.M., van der Oost J. (2003). The unique features of glycolytic pathways in Archaea. Biochem. J..

[67] Siebers B., Schönheit P. (2005). Unusual pathways and enzymes of central carbohydrate metabolism in Archaea. Curr. Opin. Microbiol..

[68] Buchholz S.E., Dooley M.M., Eveleigh D.E. (1987). *Zymomonas*—An alcoholic enigma. Trends Biotechnol..

[69] Canale-Parola E. (1970). Biology of the sugar-fermenting *Sarcinae*. Microbiol. Mol. Biol. Rev..

[70] Duggleby R.G. (2006). Domain relationships in thiamine diphosphate-dependent enzymes. Acc. Chem. Res..

[71] Costelloe S., Ward J., Dalby P. (2008). Evolutionary analysis of the TPP-dependent enzyme family. J. Mol. Evol..

[72] Kluger R., Tittmann K. (2008). Thiamin diphosphate catalysis: Enzymic and nonenzymic covalent intermediates. Chem. Rev..

[73] Schellenberger A. (1998). Sixty years of thiamin diphosphate biochemistry. Biochim. Biophys. Acta.

[74] Iding H., Siegert P., Mesch K., Pohl M. (1998). Application of alpha-keto acid decarboxylases in biotransformations. Biochim. Biophys. Acta.

[75] Bringer-Meyer S., Schimz K.L., Sahm H. (1986). Pyruvate decarboxylase from *Zymomonas mobilis*. Isolation and partial characterization. Arch. Microbiol..

[76] Raj K.C., Ingram L.O., Maupin-Furlow J.A. (2001). Pyruvate decarboxylase: A key enzyme for the oxidative metabolism of lactic acid by *Acetobacter pasteurianus*. Arch. Microbiol..

[77] Raj K.C., Talarico L.A., Ingram L.O., Maupin-Furlow J.A. (2002). Cloning and characterization of the *Zymobacter palmae* pyruvate decarboxylase gene (*pdc*) and comparison to bacterial homologues. Appl. Environ. Microbiol..

[78] Talarico L.A., Ingram L.O., Maupin-Furlow J.A. (2001). Production of the Gram-positive *Sarcina ventriculi* pyruvate decarboxylase in *Escherichia coli*. Microbiology.

[79] Wang Q., He P., Lu D., Shen A., Jiang N. (2004). Purification, characterization, cloning and expression of pyruvate decarboxylase from *Torulopsis glabrata* IFO005. J. Biochem. (Tokyo).

[80] Ma K., Hutchins A., Sung S.-J.S., Adams M.W.W. (1997). Pyruvate ferredoxin oxidoreductase from the hyperthermophilic archaeon, *Pyrococcus furiosus*, functions as a CoA-dependent pyruvate decarboxylase. Proc. Natl. Acad. Sci. USA.

[81] Dobritzsch D., Konig S., Schneider G., Lu G. (1998). High resolution crystal structure of pyruvate decarboxylase from *Zymomonas mobilis*. Implications for substrate activation in pyruvate decarboxylases. J. Biol. Chem..

[82] Jordan F. (2003). Current mechanistic understanding of thiamin diphosphate-dependent enzymatic reactions. Nat. Prod. Rep..

[83] Kluger R. (1987). Thiamin diphosphate: A mechanistic update on enzymic and nonenzymic catalysis of decarboxylation. Chem. Rev..

[84] Candy J.M., Duggleby R.G. (1998). Structure and properties of pyruvate decarboxylase and site-directed mutagenesis of the *Zymomonas mobilis* enzyme. Biochim. Biophys. Acta.

[85] Kutter S., Weiss M.S., Wille G., Golbik R., Spinka M., König S. (2009). Covalently bound substrate at the regulatory site of yeast pyruvate decarboxylases triggers allosteric enzyme activation. J. Biol. Chem..

[86] Siegert P., McLeish M.J., Baumann M., Iding H., Kneen M.M., Kenyon G.L., Pohl M. (2005). Exchanging the substrate specificities of pyruvate decarboxylase from *Zymomonas mobilis* and benzoylformate decarboxylase from *Pseudomonas putida*. Protein Eng. Des. Sel..

[87] rjunan P., Umland T., Dyda F., Swaminathan S., Furey W., Sax M., Farrenkopf B., Gao Y., Zhang D., Jordan F. (1996). Crystal structure of the thiamin diphosphate-dependent enzyme pyruvate decarboxylase from the yeast *Saccharomyces cerevisiae* at 2.3 Å resolution. J. Mol. Biol..

[88] Pohl M. (1997). Protein design on pyruvate decarboxylase (PDC) by site-directed mutagenesis. Adv. Biochem. Eng. Biotechnol..

[89] Liu M., Sergienko E.A., Guo F., Wang J., Tittmann K., Hubner G., Furey W., Jordan F. (2001). Catalytic acid-base groups in yeast pyruvate decarboxylase. 1. Site-directed mutagenesis and steady-state kinetic studies on the enzyme with the D28A, H114F, H115F, and E477Q substitutions. Biochemistry (Mosc.).

[90] Schenk G., Layfield R., Candy J.M., Duggleby R.G., Nixon P.F. (1997). Molecular evolutionary analysis of the thiamine-diphosphate-dependent enzyme, transketolase. J. Mol. Evol..

[91] Raeburn S., Rabinowitz J.C. (1971). Pyruvate: Ferredoxin oxidoreductase: II. Characteristics of the forward and reverse reactions and properties of the enzyme. Arch. Biochem. Biophys..

[92] Uyeda K., Rabinowitz J.C. (1971). Pyruvate-ferredoxin oxidoreductase. IV. Studies on the reaction mechanism. J. Biol. Chem..

[93] Uyeda K., Rabinowitz J.C. (1971). Pyruvate-ferredoxin oxidoreductase. III. Purification and properties of the enzyme. J. Biol. Chem..

[94] Ragsdale S.W. (2003). Pyruvate ferredoxin oxidoreductase and its radical intermediate. Chem. Rev..

[95] Ragsdale S.W., Pierce E. (2008). Acetogenesis and the Wood-Ljungdahl pathway of CO_2_ fixation. Biochim. Biophys. Acta.

[96] Tittmann K. (2009). Reaction mechanisms of thiamin diphosphate enzymes: Redox reactions. FEBS J..

[97] Kletzin A., Adams M. (1996). Molecular and phylogenetic characterization of pyruvate and 2- ketoisovalerate ferredoxin oxidoreductases from *Pyrococcus furiosus* and pyruvate ferredoxin oxidoreductase from *Thermotoga maritima*. J. Bacteriol..

[98] Zhang Q., Iwasaki T., Wakagi T., Oshima T. (1996). 2-Oxoacid: Ferredoxin oxidoreductase from the thermoacidophilic archaeon, *Sulfolobus* sp. strain 7. J. Biochem. (Tokyo).

[99] Townson S.M., Upcroft J.A., Upcroft P. (1996). Characterisation and purification of pyruvate: Ferredoxin oxidoreductase from *Giardia duodenalis*. Mol. Biochem. Parasitol..

[100] Horner D.S., Hirt R.P., Embley T.M. (1999). A single eubacterial origin of eukaryotic pyruvate: Ferredoxin oxidoreductase genes: Implications for the evolution of anaerobic eukaryotes. Mol. Biol. Evol..

[101] Pineda E., Encalada R., Rodríguez-Zavala J.S., Olivos-García A., Moreno-Sánchez R., Saavedra E. (2010). Pyruvate: Ferredoxin oxidoreductase and bifunctional aldehyde-alcohol dehydrogenase are essential for energy metabolism under oxidative stress in *Entamoeba histolytica*. FEBS J..

[102] Wahl R.C., Orme-Johnson W.H. (1987). Clostridial pyruvate oxidoreductase and the pyruvate-oxidizing enzyme specific to nitrogen fixation in *Klebsiella pneumoniae* are similar enzymes. J. Biol. Chem..

[103] Meinecke B., Bertram J., Gottschalk G. (1989). Purification and characterization of the pyruvate-ferredoxin oxidoreductase from *Clostridium acetobutylicum*. Arch. Microbiol..

[104] Pieulle L., Magro V., Hatchikian E. (1997). Isolation and analysis of the gene encoding the pyruvate-ferredoxin oxidoreductase of *Desulfovibrio africanus*, production of the recombinant enzyme in *Escherichia coli*, and effect of carboxy-terminal deletions on its stability. J. Bacteriol..

[105] Pieulle L., Guigliarelli B., Asso M., Dole F., Bernadac A., Hatchikian E.C. (1995). Isolation and characterization of the pyruvate-ferredoxin oxidoreductase from the sulfate-reducing bacterium *Desulfovibrio africanus*. Biochim. Biophys. Acta.

[106] Pieulle L., Charon M.-H., Bianco P., Bonicel J., Petillot Y., Hatchikian E.C. (1999). Structural and kinetic studies of the pyruvate-ferredoxin oxidoreductase/ferredoxin complex from *Desulfovibrio africanus*. Eur. J. Biochem..

[107] Pieulle L., Chabriere E., Hatchikian C., Fontecilla-Camps J.C., Charon M.-H. (1999). Crystallization and preliminary crystallographic analysis of the pyruvate-ferredoxin oxidoreductase from *Desulfovibrio africanus*. Acta Crystallogr. D Biol. Crystallogr..

[108] Blamey J.M., Adams M.W. (1994). Characterization of an ancestral type of pyruvate ferredoxin oxidoreductase from the hyperthermophilic bacterium, *Thermotoga maritima*. Biochemistry (Mosc.).

[109] Blamey J.M., Adams M.W.W. (1993). Purification and characterization of pyruvate ferredoxin oxidoreductase from the hyperthermophilic archaeon *Pyrococcus furiosus*. Biochim. Biophys. Acta.

[110] Kunow J., Linder D., Thauer R.K. (1995). Pyruvate: Ferredoxin oxidoreductase from the sulfate-reducing *Archaeoglobus fulgidus*: Molecular composition, catalytic properties, and sequence alignments. Arch. Microbiol..

[111] Bock A.-K., Prieger-Kraft A., Schönheit P. (1994). Pyruvate a novel substrate for growth and methane formation in *Methanosarcina barkeri*. Arch. Microbiol..

[112] Bock A.K., Kunow J., Glasemacher J., Schönheit P. (1996). Catalytic properties, molecular composition and sequence alignments of pyruvate: Ferredoxin oxidoreductase from the methanogenic archaeon *Methanosarcina Barkeri* (Strain Fusaro). Eur. J. Biochem..

[113] Tersteegen A., Dietmar L.R.K., Thauer R.H. (1997). Structures and functions of four anabolic 2-oxoacid oxidoreductases in *Methanobacterium thermoautotrophicum*. Eur. J. Biochem..

[114] Chabrière E., Vernede X., Guigliarelli B., Charon M.-H., Hatchikian E.C., Fontecilla-Camps J.C. (2001). Crystal structure of the free radical intermediate of pyruvate:ferredoxin oxidoreductase. Science.

[115] Chabrière E., Charon M.-H., Volbeda A., Pieulle L., Hatchikian E.C., Fontecilla-Camps J.-C. (1999). Crystal structures of the key anaerobic enzyme pyruvate: Ferredoxin oxidoreductase, free and in complex with pyruvate. Nat. Struct. Mol. Biol..

[116] Garczarek F., Dong M., Typke D., Witkowska H.E., Hazen T.C., Nogales E., Biggin M.D., Glaeser R.M. (2007). Octomeric pyruvate-ferredoxin oxidoreductase from *Desulfovibrio vulgaris*. J. Struct. Biol..

[117] Bock A.-K., Schönheit P., Teixeira M. (1997). The iron-sulfur centers of the pyruvate:ferredoxin oxidoreductase from *Methanosarcina barkeri* (Fusaro). FEBS Lett..

[118] Charon M.-H., Volbeda A., Chabriere E., Pieulle L., Fontecilla-Camps J.C. (1999). Structure and electron transfer mechanism of pyruvate: Ferredoxin oxidoreductase. Curr. Opin. Struct. Biol..

[119] Shiba H., Kawasumi T., Igarashi Y., Kodama T., Minoda Y. (1985). The CO_2_ assimilation via. the reductive tricarboxylic acid cycle in an obligately autotrophic, aerobic hydrogen-oxidizing bacterium, *Hydrogenobacter thermophilus*. Arch. Microbiol..

[120] Ikeda T., Ochiai T., Morita S., Nishiyama A., Yamada E., Arai H., Ishii M., Igarashi Y. (2006). Anabolic five subunit-type pyruvate: Ferredoxin oxidoreductase from *Hydrogenobacter thermophilus* TK-6. Biochem. Biophys. Res. Commun..

[121] Ikeda T., Yamamoto M., Arai H., Ohmori D., Ishii M., Igarashi Y. (2010). Enzymatic and electron paramagnetic resonance studies of anabolic pyruvate synthesis by pyruvate: Ferredoxin oxidoreductase from *Hydrogenobacter thermophilus*. FEBS J..

[122] Yamamoto M., Ikeda T., Arai H., Ishii M., Igarashi Y. (2010). Carboxylation reaction catalyzed by 2-oxoglutarate: Ferredoxin oxidoreductases from *Hydrogenobacter thermophilus*. Extremophiles.

[123] Lin W.C., Yang Y.-L., Whitman W.B. (2003). The anabolic pyruvate oxidoreductase from Methanococcus maripaludis. Arch. Microbiol..

[124] Lin W., Whitman W. (2004). The importance of *por*E and *por*F in the anabolic pyruvate oxidoreductase of *Methanococcus maripaludis*. Arch. Microbiol..

[125] Imlay A.J. (2006). Iron-sulphur clusters and the problem with oxygen. Mol. Microbiol..

[126] Linn T.C., Pettit F.H., Reed L.J. (1969). α-Keto acid dehydrogenase complexes, X. Regulation of the activity of the pyruvate dehydrogenase complex from beef kidney mitochondria by phosphorylation and dephosphorylation. Proc. Natl. Acad. Sci. USA.

[127] Patel M.S., Roche T.E. (1990). Molecular biology and biochemistry of pyruvate dehydrogenase complexes. FASEB J..

[128] Witzmann S., Bisswanger H. (1998). The pyruvate dehydrogenase complex from thermophilic organisms: Thermal stability and re-association from the enzyme components. Biochim. Biophys. Acta.

[129] Potter S., Fothergill-Gilmore L.A. (1992). Purification and properties of pyruvate kinase from *Thermoplasma acidophilum*. FEMS Microbiol. Lett..

[130] Heath C., Jeffries A.C., Hough D.W., Danson M.J. (2004). Discovery of the catalytic function of a putative 2-oxoacid dehydrogenase multienzyme complex in the thermophilic archaeon *Thermoplasma acidophilum*. FEBS Lett..

[131] Selig M., Schönheit P. (1994). Oxidation of organic compounds to CO_2_ with sulfur or thiosulfate as electron acceptor in the anaerobic hyperthermophilic archaea *Thermoproteus tenax* and *Pyrobaculum islandicum* proceeds via. the citric acid cycle. Arch. Microbiol..

[132] Ma K., Weiss R., Adams M.W.W. (2000). Characterization of hydrogenase II from the hyperthermophilic archaeon *Pyrococcus furiosus* and assessment of its role in sulfur reduction. J. Bacteriol..

[133] Jenney F.E., Adams M.W.W. (2008). Hydrogenases of the model hyperthermophiles. Ann. NY Acad. Sci..

[134] Rudolph F.B., Purich D.L., Fromm H.J. (1968). Coenzyme A-linked aldehyde dehydrogenase from *Escherichia coli*. J. Biol. Chem..

[135] Lurz R., Mayer F., Gottschalk G. (1979). Electron microscopic study on the quaternary structure of the isolated particulate alcohol-acetaldehyde dehydrogenase complex and on its identity with the polygonal bodies of *Clostridium kluyveri*. Arch. Microbiol..

[136] Nair R.V., Bennett G.N., Papoutsakis E.T. (1994). Molecular characterization of an aldehyde/alcohol dehydrogenase gene from *Clostridium acetobutylicum* ATCC 824. J. Bacteriol..

[137] Tóth J., Ismaiel A.A., Chen J.-S. (1999). The *ald* gene, encoding a coenzyme A-acylating aldehyde dehydrogenase, distinguishes *Clostridium beijerinckii* and two other solvent-producing clostridia from *Clostridium acetobutylicum*. Appl. Environ. Microbiol..

[138] Sánchez L.B. (1998). Aldehyde dehydrogenase (CoA-acetylating) and the mechanism of ethanol formation in the amitochondriate protist, *Giardia lamblia*. Arch. Biochem. Biophys..

[139] Bruchhaus I., Tannich E. (1994). Purification and molecular characterization of the NAD(+)-dependent acetaldehyde/alcohol dehydrogenase from *Entamoeba histolytica*. Biochem. J..

[140] Burdette D., Zeikus J.G. (1994). Purification of acetaldehyde dehydrogenase and alcohol dehydrogenases from *Thermoanaerobacter ethanolicus* 39E and characterization of the secondary-alcohol dehydrogenase (2 degrees Adh) as a bifunctional alcohol dehydrogenase--acetyl-CoA reductive thioesterase. Biochem. J..

[141] Brown S.D., Guss A.M., Karpinets T.V., Parks J.M., Smolin N., Yang S., Land M.L., Klingeman D.M., Bhandiwad A., Rodriguez M. (2011). Mutant alcohol dehydrogenase leads to improved ethanol tolerance in *Clostridium thermocellum*. Proc. Natl. Acad. Sci. USA.

